# Cellulosic ethanol production via consolidated bioprocessing at 75 °C by engineered *Caldicellulosiruptor bescii*

**DOI:** 10.1186/s13068-015-0346-4

**Published:** 2015-10-06

**Authors:** Daehwan Chung, Minseok Cha, Elise N. Snyder, James G. Elkins, Adam M. Guss, Janet Westpheling

**Affiliations:** Department of Genetics, University of Georgia, Athens, GA USA; The BioEnergy Science Center, Oak Ridge National Laboratory, Oak Ridge, TN USA; Biosciences Division, Oak Ridge National Laboratory, Oak Ridge, TN USA

**Keywords:** Cellulosic ethanol, Metabolic engineering, *Caldicellulosiruptor bescii*, Alcohol dehydrogenase, *Thermoanaerobacter pseudethanolicus* 39E

## Abstract

**Background:**

The *C. bescii* genome does not encode an acetaldehyde/alcohol dehydrogenase or an acetaldehyde dehydrogenase and no ethanol production is detected in this strain. The recent introduction of an NADH-dependent AdhE from *C. thermocellum* (Fig. 1a) in an *ldh* mutant of this strain resulted in production of ethanol from un-pretreated switchgrass, but the thermolability of the *C. thermocellum* AdhE at the optimum growth temperature of *C. bescii* (78 °C) meant that ethanol was not produced above 65 °C.

**Fig. 1:**
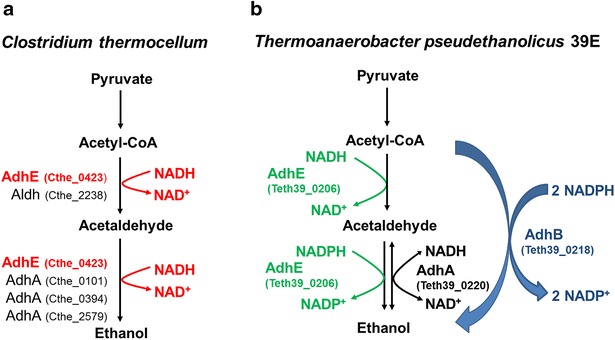
Proposed scheme for the pyruvate to ethanol pathway in *C. thermocellum* and *T. pseudethanolicus* 39E. **a** The *C. thermocellum* ethanol pathway. The *red colored* AdhE (Cthe_0423) is already expressed and tested in *C. bescii* [26]. **b** The *T. pseudethanolicus* 39E ethanol pathway. The *green colored* AdhE (Teth39_0206) and *blue colored* AdhB (Teth39_0218) are expressed and tested in *C. bescii* in this study

**Results:**

The *adhB* and *adhE* genes from *Thermoanaerobacter pseudethanolicus* 39E, an anaerobic thermophile that produces ethanol as a major fermentation product at 70 °C, were cloned and expressed in an *ldh* deletion mutant of *C. bescii*. The engineered strains produced ethanol at 75 °C, near the ethanol boiling point. The AdhB expressing strain produced ethanol (1.4 mM on Avicel, 0.4 mM on switchgrass) as well as acetate (13.0 mM on Avicel, 15.7 mM on switchgrass). The AdhE expressing strain produced more ethanol (2.3 mM on Avicel, 1.6 mM on switchgrass) and reduced levels of acetate (12.3 mM on Avicel, 15.1 mM on switchgrass). These engineered strains produce cellulosic ethanol at the highest temperature of any microorganism to date. In addition, the addition of 40 mM MOPS to the growth medium increased the maximal growth yield of *C. bescii* by approximately twofold.

**Conclusions:**

The utilization of thermostable enzymes will be critical to achieving high temperature CBP in bacteria such as *C. bescii*. The ability to produce ethanol at 75 °C, near its boiling point, raises the possibility that process optimization could allow in situ product removal of this end product to mitigate ethanol toxicity.

**Electronic supplementary material:**

The online version of this article (doi:10.1186/s13068-015-0346-4) contains supplementary material, which is available to authorized users.

## Background

Production of renewable and sustainable biofuels from lignocellulosic biomass using thermophilic bacteria has attracted increasing attention because of the potential advantages of using high temperature fermentation, including improved kinetics, increased mixing rate, lower oxygen solubility, reduced risk of microbial contamination, reduced costs for cooling and heating during the bioconversion process, and providing the possibility of ethanol separation in continuous cultures [1–6]. Fermentative thermophilic anaerobic bacteria have been used to convert plant biomass into ethanol without added lignocellulose-degrading enzymes using a process called consolidated bioprocessing (CBP) [7–9]. Much of the CBP research to date has focused on *Clostridium thermocellum* [10–12], which grows at 60 °C and produces ethanol, acetate, lactate, formate, CO_2_, H_2_, and amino acids as major fermentation products [8, 13]. Increased ethanol production and tolerance of this microorganism have been achieved by extensive metabolic engineering [14–21]. However, while *C. thermocellum* has the ability to deconstruct crystalline cellulose and catabolize hexose sugars, it cannot utilize hemicellulosic pentose sugars such as xylose and arabinose for growth and its optimal growth temperature is 60 °C [8, 12]. To fully realize the potential of thermophilic CBP, several major improvements over the state of the art will be needed, including increased ethanol yield and titer, co-utilization of hemicellulosic sugars, and ethanol production near or above the boiling point of ethanol (~78 °C).

*Caldicellulosiruptor bescii*, previously known as “*Anaerocellum thermophilum*”, is an anaerobic, Gram-positive bacterium originally isolated from the hot springs of Kamchatka, Russia [22–24]. This microorganism has been reported to grow on cellulose up to 83 °C with an optimum temperature of 78 °C at pH 7 and is among the most thermophilic cellulolytic organisms discovered to date [23, 24]. It is able to efficiently ferment crystalline cellulose, hemicellulosic sugars, xylan, and un-pretreated plant biomass, such as hardwood poplar, switchgrass and Napier grasses, producing acetate, lactate, and H_2_ [12, 23]. These characteristics could provide unique advantages over existing CBP approaches, avoiding costly lignocellulose pretreatment and allowing separation of ethanol from continuously growing cultures [25, 26].

Recently, new genetic tools have been successfully used for insertion, deletion, and heterologous/homologous expression of genes in *C. bescii* [26–29]. Like other near-maximal H_2_-yielding anaerobic microbes, *C. bescii* does not encode either AdhE (bi-functional acetaldehyde/alcohol dehydrogenase) or ALDH (acetaldehyde dehydrogenase) [30]; thus, no ethanol production is detected in this strain [23]. However, its chromosome encodes two AdhAs (alcohol dehydrogenase), Cbes0928 and Cbes0224 [31]. Introduction of a heterologous ethanol production pathway via expression of the NADH-dependent AdhE from *C. thermocellum* (Fig. 1a) in a *C. bescii ldh* mutant strain resulted in production of ethanol from both model substrates and un-pretreated switchgrass [26]. Although neither the ethanol yield nor titer was sufficient to meet requirements for economic product recovery, the bioethanol production directly from real-world substrates opens the door to development of the next generation of CBP. In addition, because of the insufficient thermostability of the *C. thermocellum* AdhE, this engineered strain was only able to produce ethanol up to 65 °C [26], which is far below the *C. bescii* optimum growth temperature and the ethanol boiling temperature.

To overcome this issue, we sought to identify a more thermostable ethanol pathway to introduce into *C. bescii.T. pseudethanolicus* 39E is a Gram-positive, anaerobic thermophile that produces ethanol as a major fermentation product and grows up to 70 °C [32–34]. It encodes two bi-functional alcohol dehydrogenases, *adhB* and *adhE* [4, 35]. The bi-functional acetyl-CoA thioesterase/alcohol dehydrogenase (AdhB) is NADPH-dependent and proposed as a key terminal enzyme for ethanol production in *T. pseudethanolicus* based on microarray data [4, 32]. The other bi-functional acetaldehyde/alcohol dehydrogenase (AdhE) showed NADH-dependent ALDH activity and weak NADPH-dependent ADH activity [32, 36]. Utilization of more thermostable enzymes such as these will be critical to achieving high temperature CBP in *C. bescii*. Here, we explore the use of these more thermostable enzymes from *T. pseudethanolicus* to develop ethanol production pathways that function near the optimal growth temperature in *C. bescii*. Further, we explore optimization of culture medium buffering capacity as a means of improving growth and fermentation in engineered *C. bescii* strains under batch conditions.

## Results and discussion

### Higher buffering capacity of culture medium improves *C. bescii* growth

**Fig. 2:**
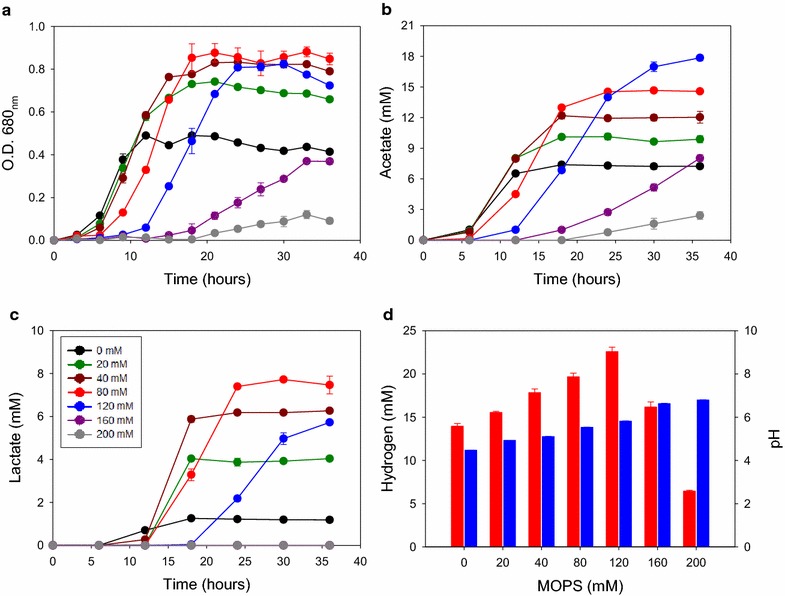
Effect of buffer (MOPS) concentrations during the cultivation of wild-type *C. bescii* in closed-serum bottles without pH control. **a** Growth properties measured by optical density at 680 nm, **b** acetate production, and **c** lactate production as a function of increasing MOPS concentration. *Black*, 0 mM; *green*, 20 mM; *dark red*, 40 mM; *light red*, 80 mM; *blue*, 120 mM; *purple*, 160 mM, *gray*, 200 mM. **d** Final pH of media (*blue bars*) and hydrogen production (*red bars*) after 36 h cultivation

The pH of cultures during growth is important for anaerobic microorganisms, particularly at higher growth yields [37, 38]. *C. bescii* has a narrow optimal pH range, pH 6.8–7.3, in culture media in closed bottles [24, 39] and the production of acetic and lactic acid have been shown to inhibit growth, especially at late growth phases [23, 37]. While fed-batch cultivation with pH control in a fermentor, such as that used in industrial processes, can solve this problem, pH control in bench-scale genetic experiments is not practical [40]. An alternative approach is to use high initial buffering capacity to prevent excessive pH changes [41, 42]. This has enabled higher growth yields and rates in other microorganisms [43, 44]. To systematically investigate the influence of increased buffer concentrations on the growth yield, metabolite production, and final pH values of *C. bescii* fermentations; 3-(*N*-morpholino) propane sulfonic acid (MOPS) was used as a buffering reagent in addition to the 2.4 mM phosphate normally used in this medium supplemented with 1 % cellobiose as the carbon source [39]. Growth was tested with MOPS concentrations ranging from 0 to 200 mM to determine if pH was limiting bioconversion (Fig. 2). At 160 mM MOPS, growth was severely inhibited, and virtually no growth was observed at 200 mM MOPS. At lower MOPS concentrations, however, a clear improvement was observed. The maximal growth yield of *C. bescii* increased approximately twofold as measured by optical density at 680 nm (OD_680_) when the MOPS concentration in the medium was increased from 0 to 40 mM (Fig. 2a). At 80 and 120 mM MOPS, a similar final OD was attained, but only after a lag period. Because *C. bescii* can grow at relatively high osmotic pressure (>550 mOsmol) [37, 45], the longer lag phase at MOPS concentrations of 80 mM and above suggests a mechanism other than osmotic stress to explain this difference (Fig. 2a) [40, 46]. The final pH of the culture medium increased from 4.5 to 5.8 as the MOPS concentration was increased from 0 to 120 mM (Fig. 2d). These results are consistent with previous results in pH-controlled fermenters; the decrease in pH during fermentation is a limiting factor for growth and limits the ability to reach higher cell densities [37]. Substrate conversion into products also increased with MOPS concentration (Fig. 2b–d). H_2_ (Fig. 2d) and acetate production (Fig. 2b) showed a steady increase with increased buffer concentrations up to 120 mM MOPS. H_2_ production increased 62 % (from 13.9 to 22.6 mM) and acetate production increased 149 % (from 7.2 to 17.9 mM) on 120 mM MOPS compared to the culture without MOPS. The maximum yield of lactate was obtained on 80 mM MOPS (Fig. 2c). In this analysis, 40 mM MOPS was sufficient to sustain exponential growth to maximal growth yield and the fastest doubling time (~2.3 h) with a final pH ~5.1. We, therefore, used 40 mM MOPS for subsequent analyses.

### Heterologous expression of the thermostable AdhE and AdhB from *T. pseudethanolicus* in *C. bescii*

**Fig. 3:**
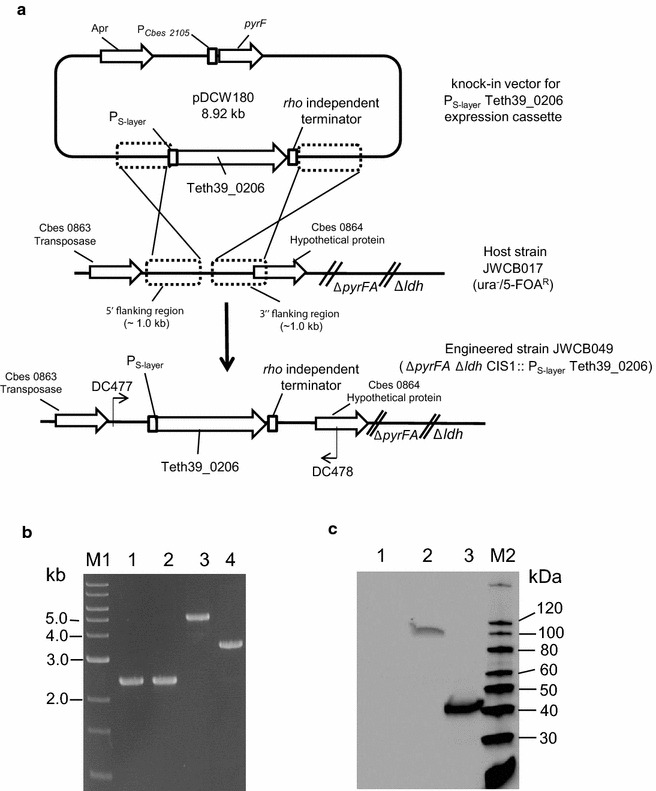
Targeted insertion and expression of the *T. pseudethanolicus* 39E *adhE* (Teth39_0206) and *adhB* (Teth39_0218) in *C. bescii*. **a** A diagram of the integration vector pDCW180 (Additional file 1: Figure S1), which contains the P_S-layer_Teth39_0206 expression cassette and *pyrF* cassette [27] for selection of transformants. Homologous recombination can occur at the *upstream*- or *downstream*-targeted chromosomal regions (inter-cistronic region between Cbes0863 and Cbes0864), integrating the plasmid into the genome and generating uracil prototroph strain. Counter selection with 5-FOA selects for loss of the plasmid sequences but not the *adhE* expression cassette. *Bent arrows* depict primers used for verification of the integrated expression cassette. *Apr* apramycin resistance gene cassette. **b** Gel depicting PCR products amplified from the targeted chromosome region in JWCB001 (wild-type; *lane 1),* JWCB017 (*ΔpyrFA Δldh*; *lane 2*), JWCB049 (*ΔpyrFA Δldh* CIS1:: P_S-layer_Teth39_0206; *lane 3*), and JWCB054 (*ΔpyrFA Δldh* CIS1:: P_S-layer_Teth39_0218; *lane 4*) amplified by primers DC477 and DC478. M1: 1-kb DNA ladder (New England Biolabs). **c** Total cell protein lysates (75 µg) isolated from mid-log phase cultures grown at 75 °C were electrophoresed in SDS-PAGE gels, followed by Western blot analysis probed with anti-His-tag antibody as described in “Methods”. *Lane 1*: JWCB017; *lane 2*: JWCB049; *lane 3*: JWCB054; M2: MagicMark™ XP Western Protein Standard (Invitrogen)

The *T. pseudethanolicus adhE* gene (bifunctional secondary ADH/aldehyde hydrogenase, Teth39_0206) and *adhB* gene (bifunctional secondary ADH/Acetyl-CoA thioesterase, Teth39_0218) were cloned from *T. pseudethanolicus* 39E under the transcriptional control of the *C. bescii* S-layer protein (Cbes2303) promoter (P_S-layer_) [26, 29] (Additional file 1: Figures S1, S2; Table 1). Both the P_S-layer_Teth39_0206 (*adhE*) and P_S-layer_Teth39_0218 (*adhB*) expression cassettes contain a Rho-independent transcription terminator and C-terminal 6X His-tag to facilitate protein purification and Western blotting. These genes were each inserted into the inter-cistronic region between Cbes0863 and Cbes0864 of the chromosome in *C. bescii* strain JWCB017 (*ΔpyrFA Δldh*) (Fig. 3a). Uracil prototrophic transformants were serially passaged as described [26] to allow segregation of merodiploids containing a mixture of the integrated *adhE* expression cassette and the wild-type genomes. This resulted in strains JWCB049 (*ΔpyrFA Δldh* CIS1:: P_S-layer_Teth39_0206), and JWCB054 (*ΔpyrFA Δldh* CIS1:: P_S-layer_Teth39_0218) (Table 1). Verification of JWCB049 and JWCB054 was performed using PCR amplification with primers DC477 and DC478 (Fig. 3b), and sequencing the PCR products. The parent strain, JWCB017, produced the expected wild type 2.44-kb band, whereas amplification of JWCB049 and JWCB054 produced ~5.0-kb and ~ 3.5-kb bands, respectively, indicating an insertion of the *adhE* and *adhB* expression cassettes within the targeted region (Fig. 3b). The previous deletion of the *ldh* gene in JWCB017, JWCB049, and JWCB054 was also confirmed (Additional file 1: Figure S3).

**Table 1:** Strain and plasmids used in this study

Strains	Strain and genotype/phenotype	Source
*Escherichia coli*		
JW338	*DH5α* containing pDCW180 (Apramycin^R^)	This study
JW341	*DH5α* containing pDCW183 (Apramycin^R^)	This study
*Caldicellulosiruptor bescii*		
JWCB001	wild type/(ura^+^/5-FOA^S^)	DSMZ^a^
JWCB017	*ΔpyrFAΔldh*/(ura^−^/5-FOA^R^)	[49]
JWCB049	*ΔpyrFA Δldh* CIS1:: P_S-layer_Teth39_0206 (*adhE*)/(ura^−^/5-FOA^R^)	This study
JWCB054	*ΔpyrFA Δldh* CIS1:: P_S-layer_Teth39_0218 (*adhB*)/(ura^−^/5-FOA^R^)	This study
Plasmids		
pDCW142	Intermediate vector (Apramycin^R^)	[26]
pDCW180	Integration vector containing P_S-layer_Teth39*_*0206 (Apramycin^R^)	This study
pDCW183	Integration vector containing P_S-layer_Teth39*_*0218 (Apramycin^R^)	This study

^a^Deutsche Sammlung von Mikroorganismen und Zellkulturen

To test expression of the *T. pseudethanolicus* AdhE and AdhB proteins in *C. bescii*, JWCB049 and JWCB054 were grown in low osmolarity defined (LOD) medium [39] to mid-log phase at 75 °C (Fig. 3c). Cell extracts were then examined by Western hybridization using monoclonal anti-His antibodies (Fig. 3c). An approximately 100 kDa protein from the AdhE strain (lane 2 in Fig. 3c) and an approximately 40 kDa protein from the AdhB strain (lane 3 in Fig. 3c) were detected, which correspond to the predicted molecular weights for the His-tagged proteins. No proteins were detected by the anti-His antibodies in the parental strain (JWCB017, lane 1 in Fig. 3c). Interestingly, the AdhE protein (lane 2) was less abundant than AdhB (lane 3). The AdhE protein contains a linker region between the ALDH and ADH domains [47] and this kind of linker region has been observed to be susceptible to proteolysis in various multi-domain proteins, which may explain the lower abundance of *T. pseudethanolicus* AdhE relative to AdhB in *C. bescii*. Alternatively, even small differences in *T. pseudethanolicus* codon usage could decrease translational efficiency in *C. bescii*, which would suggest codon optimization as a future strategy to increase expression.

### Engineered *C. bescii* strains produce ethanol from cellobiose at 75 °C

**Fig. 4:**
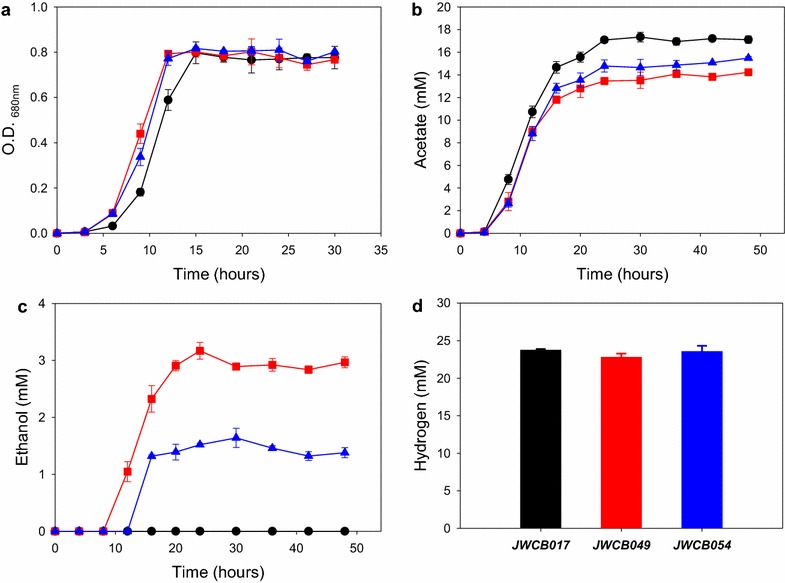
Growth and product formation of the mutant strains on cellobiose. **a** Growth properties measured by optical density (OD_680nm_), **b** acetate production, and **c** ethanol production of *C. bescii* mutant strains on 1 % cellobiose as a sole carbon source at 75 °C; *black circles*, JWCB017; *red squares*, JWCB049; *blue triangles*, JWCB054. **d** Hydrogen produced by each strain at the end of fermentation (48 h); *black bars*, JWCB017; *red bars*, JWCB049; *blue bars*, JWCB054. *Error bars* based on two biologically independent experiments

In previous work to engineer ethanol production in *C. bescii*, mutation of the lactate dehydrogenase gene (*ldh*) was obtained in the *C. bescii* chromosome [48] and an *adhE* gene from *C. thermocellum* was introduced [26]. Deletion of the *ldh* gene resulted in a strain, JWCB017 that produced no lactate and more acetate and hydrogen than the wild type [49]. Addition of the *adhE* gene to the *ldh* mutant strain resulted in a strain that produced ethanol and less acetate, presumably because carbon was channeled from acetate to ethanol [26]. The introduction of new fermentation pathways could affect growth and product formation due to potential redox imbalances. To investigate the functionality of *T.pseudethanolicus* AdhE and AdhB in *C. bescii*, strains were characterized in LOD medium supplemented with 40 mM MOPS and 1 % (wt/vol) cellobiose. We first compared growth rate of the strains expressing AdhE (JWCB049) and AdhB (JWCB054) to the parent strain (JWCB017) at 75 °C (Fig. 4). There was no obvious difference in growth rate, with calculated doubling times of 2.0, 1.9, and 1.9 h for JWCB017, JWCB049 and JWCB054, respectively. Further, all three strains reached similar maximal optical densities (~0.8 at OD_680 nm_) after 15 h incubation (Fig. 4a), suggesting that heterologous expression of these genes is not detrimental to *C. bescii*.

Fermentation products were also monitored into the stationary growth phase by high performance liquid chromatography (HPLC) and gas chromatography (Fig. 4b–d), and final product yields were calculated (Table 2). As expected, none of the strains produced lactate because they contain a deletion of the lactate dehydrogenase gene. *C. bescii* JWCB017 (*ΔpyrFA Δldh)* produced 17.0 mM acetate, 23.9 mM H_2_, and no ethanol (Fig. 4b, c), representing 12.9 % conversion of cellobiose into acetate and 91 % carbon recovery overall without accounting for microbial cell mass. The AdhB expressing strain JWCB054 produced 1.4 mM ethanol in addition to 15.5 mM acetate and 23.2 mM H_2_ from cellobiose (Fig. 4b–d), representing 11.6 % conversion of cellobiose into acetate and 1.1 % into ethanol with an overall 88 % carbon recovery (Table 2). The AdhE expressing strain JWCB049 produced approximately twice as much ethanol (2.9 mM) as strain JWCB054 and showed reduced production of acetate (14.1 mM) and H_2_ (22.5 mM) (Fig. 4b, c; Table 2) compared to the parental stain JWCB017, indicating redirection of carbon flow from pyruvate away from acetate and toward ethanol. JWCB049 showed 10.7 % conversion of cellobiose into acetate and 2.2 % to ethanol with 81 % overall carbon recovery. Interestingly, H_2_ production is reduced in strain JWCB049, but not in strain JWCB054. This may be explained by the fact that H_2_ production in *C. bescii* is likely catalyzed by an NADH- and ferredoxin-dependent electron bifurcating [FeFe] hydrogenase (Cbes1297-1299) [50]. Thus, the AdhE pathway, which utilizes one NADH and one NADPH, partially competes with the hydrogenase for electrons, while the strictly NADPH-dependent AdhB does not.

**Table 2:** Carbon balance of fermentation products after 48 h fermentation

Strains	Concentration of residual compounds (at 48 h), mM									Product yieds, mol/mol			
	Cellobiose initial	Cellobiose final	Glucose	Cellobiose catablized	Lactate	Acetate	Ethanol	Hydrogen	CR (%)	*Y*_*L/C*_	*Y*_*A/C*_	*Y*_*E/C*_	*Y*_*H/C*_
JWCB017	33.1 ± 0.42	19.7 ± 0.03	17.4 ± 0.19	4.7 ± 0.27	0	17.1 ± 0.32	0.0	23.9 ± 0.15	91	0	3.64	0	5.08
JWCB049	33.1 ± 0.05	19.6 ± 0.21	16.5 ± 0.07	5.3 ± 0.12	0	14.2 ± 0.08	2.9 ± 0.09	22.5 ± 0.39	81	0	2.67	0.54	4.25
JWCB054	33.4 ± 0.14	20.5 ± 0.07	16.2 ± 0.05	4.8 ± 0.05	0	15.5 ± 0.14	1.4 ± 0.09	15.5 ± 0.77	88	0	3.23	0.28	4.83

Substrate and product concentration of wild-type, JWCB017, JWCB049 and JWCB054 are reported in mM with average percent carbon recovery (CR) and average yields in mol/mol. The fermentation conditions were as described in “Methods”. Product yields are calculated as: *Y*_*L/C*_ lactate yield per mole cellobiose, *Y*_*A/C*_ acetate yield per mole cellobiose, *Y*_*E/C*_ ethanol yield per mole cellobiose, *Y*_*H/C*_ hydrogen yield per mole cellobiose

### Engineered *C. bescii* strains produce ethanol from Avicel and lignocellulose at 75 °C

**Fig. 5:**
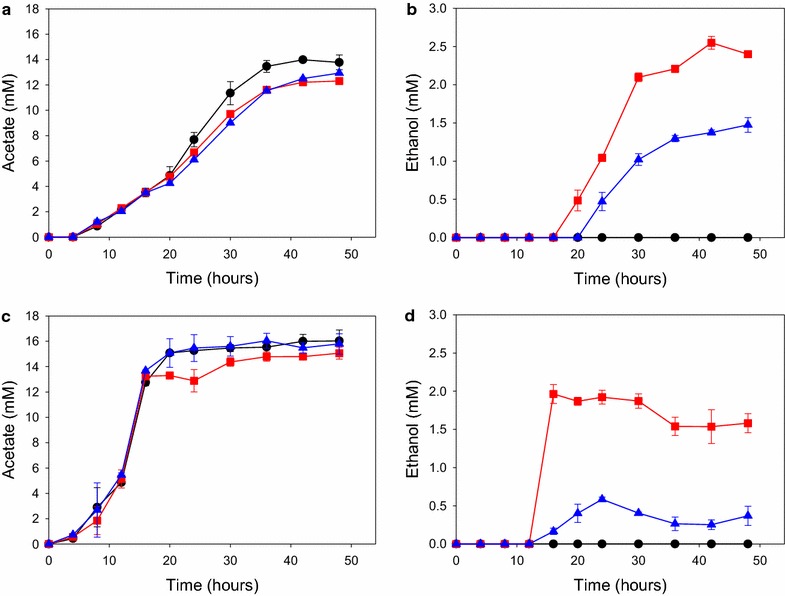
Fermentation product analysis of *C. bescii* mutant strains on Avicel and switchgrass. Analysis of fermentation products acetate (**a**, **c**), and ethanol (**b**, **d**) after growth on Avicel [2 % (wt/vol)] (**a**, **b**) and switchgrass [2 % (wt/vol)] (**c**, **d**) at 75 °C. *Black circles*, JWCB0017; *red squares*, JWCB049; *blue triangles*, JWCB054. *Error bars* based on two biologically independent experiments

To demonstrate the utility of this ethanol-producing pathway for high temperature CBP, we also investigated the ability of the engineered strains to produce ethanol from the model substrate crystalline cellulose (2 % Avicel) and the real-world cellulosic substrate switchgrass (2 %). Results were similar to those observed for cellobiose (Fig. 5). As expected, parent strain JWCB017 produced acetate (13.8 mM from Avicel, 16.1 mM from switchgrass) but no ethanol. The AdhB expressing strain, JWCB054, produced ethanol (1.4 mM on Avicel, 0.4 mM on switchgrass) as well as acetate (13.0 mM on Avicel, 15.7 mM on switchgrass). The AdhE expressing strain, JWCB049, produced more ethanol (2.3 mM on Avicel, 1.6 mM on switchgrass) than strain JWCB054 and reduced levels of acetate (12.3 mM on Avicel, 15.1 mM on switchgrass). To date, these strains represent the maximum temperature at which cellulosic ethanol production has been engineered into a microorganism.

On all substrates, cofactor specificity of the ethanol synthesis pathway likely limits ethanol formation. Previous work focused on expression of *C. thermocellum adhE* in *C. bescii* [26], which utilizes NADH as an electron donor for both acetyl-CoA reduction to acetaldehyde (ALDH activity) and acetaldehyde reduction to ethanol (ADH activity). The *T. pseudethanolicus* AdhB and AdhE enzymes, on the other hand, both utilize NADPH for ADH activity, and AdhB also utilizes NADPH for its Acetyl-CoA thioesterase activity (Fig. 1b). While diverse pathways for NADPH generation are known, the mechanism by which *C. bescii* generates NADPH is not known. *C. bescii* does not encode an obvious Glucose-6-phosphate dehydrogenase, suggesting it does not utilize the non-oxidative Pentose Phosphate Pathway (PPP), nor does it encode a malate dehydrogenase that would be necessary for a “malate shunt” as is seen in *C. thermocellum* [51]. *C. bescii* does encode a putative NADH:Ferredoxin:NADP^+^ oxidoreductase (NfnAB; Athe_0644-0645) [31, 52], that would take electrons from NADH and reduced ferredoxin to generate two NADPH, but the in vivo importance of NfnAB in *C. bescii* is unknown.

We hypothesize that low electron flux through NADPH could be limiting ethanol production. The AdhE pathway requires one NADPH per ethanol, while the AdhB pathway requires two NADPH per ethanol (Fig. 1b). If NADPH were limiting, we would expect the AdhE pathway to produce twice as much ethanol as the AdhB pathway. Indeed, this is exactly what is observed. The ethanol yield per mole of consumed cellobiose in the *adhE* expression strain was twice that of the *adhB* expression strain (0.54 and 0.28 mol ethanol/mol cellobiose for the AdhE and AdhB strains, respectively) (Table 2). Furthermore, both strains produced substantially less ethanol compared to our previous study in which we expressed the NADH-dependent *C. thermocellum* AdhE (15.3 mM) [26], again supporting the idea that NADPH availability is limiting ethanol production when utilizing these NADPH-dependent pathways in *C. bescii*. Future engineering efforts to increase NADPH availability or alter cofactor specificity have the potential to further increase ethanol yields. By producing ethanol at 75 °C, this work lays the foundations for further metabolic engineering and process optimization that could lead to in situ ethanol removal during fermentation to enhance recovery and decrease product toxicity.

## Conclusions

To gain the most value from high temperature CBP, both C5 and C6 sugars will need to be converted into fuel at high yield and titer at a temperature near or above the boiling point of ethanol. Here we report metabolically engineered strains that produce ethanol in vivo near the ethanol boiling point by introducing thermostable AdhB and AdhE proteins from *T. pseudethanolicus* into *C. bescii*. The yield and titer of ethanol are still low, but the prospect of further strain modification to improve NADPH availability or alter co-factor specificity of the enzymes has the potential to further improve upon this strain. Further, this work provides insights into mechanisms of redox balancing within *C. bescii* that will assist future metabolic engineering efforts.

## Methods

### Strains, media and culture conditions

*C. bescii* and *E. coli* strains and plasmids used in this study are listed in Table 1. All *C. bescii* strains were grown anaerobically in liquid or on solid media in low osmolality defined (LOD) medium [39], final pH 7.0, with maltose [0.5 % (wt/vol); catalog no. M5895, Sigma-Aldrich, St. Louis, MO] used as the sole carbon source for routine growth and transformation experiments. Liquid cultures were grown at 75 °C in anaerobic culture serum bottles with five gassing cycles with argon. For uracil auxotrophs, the LOD medium was supplemented with 40 µM uracil. *E. coli* strain DH5α was used for plasmid DNA construction and preparation. Standard techniques for *E. coli* were performed as described [53]. *E. coli* cells were grown in LB broth supplemented with apramycin (50 μg/mL) and plasmid DNA was isolated using a Qiagen Miniprep Kit. Chromosomal DNA from *C. bescii* strains was extracted using the Quick-gDNA MiniPrep (Zymo Research, Irvine, CA, USA) or the DNeasy Blood and Tissue Kit (Qiagen, Valencia, CA, USA) according to the manufacturer’s instructions.

### Vector construction for the knock-in of Teth39_0206 (*adhE*) and Teth39_0218 (*adhB*) into *C. besci*

The plasmids described below were generated using Q5 High-Fidelity DNA polymerase (New England BioLabs, Ipswich, MA, USA) for PCR reactions, restriction enzymes (New England BioLabs, Ipswich, MA, USA), and the Fast-link DNA Ligase kit (Epicentre Biotechnologies, Madison, WI, USA) according to the manufacturer’s instructions. Plasmid pDCW180 (Fig. 1a; Additional file 1: Figure S1) was constructed by inserting the Teth39_0206 open reading frame into pDCW142 [26], which contains the regulatory region of Cbes2303, a C-terminal 6X Histidine-tag and a Rho-independent transcription terminator. The 6.3 kb DNA fragment was amplified with primers DC464 containing a BamHI cut site and DC466 containing a SphI cut site using pDCW142 as a template. A 2.62 kb DNA fragment containing the coding sequence of Teth39_0206 was amplified with DC597 containing a BamHI site and DC598 containing a SphI site using *T. pseudethanolicus* 39E genomic DNA as a template. The two linear DNA fragments were then digested with BamHI and SphI, and ligated to construct pDCW180 (8.9 kb) (Additional file 1: Figure S1). Plasmid pDCW183 (Additional file 1: Figure S2) is identical to pDCW180 except for the cloning of Teth39_0218. To make this change, a 1.06-kb DNA fragment containing the coding sequence of Teth39_0218 was amplified by PCR using DC621 (with BamHI site) and DC622 (with SphI site) using *T. pseudethanolicus* 39E genomic DNA as a template. The 6.3-kb of backbone fragment was amplified from pDCW142 [26] by PCR using DC619 (with BamHI site) and DC620 (with SphI site). DNA sequences of the primers are shown in Additional file 1: Table S1. *E. coli* strain DH5α cells were transformed by electroporation in a 2-mm gap cuvette at 2.5 V and transformants were selected for apramycin resistance. The sequences of all plasmids were verified by Automatic sequencing (Macrogen USA, Rockville, MD, USA). All plasmids are available on request.

### *C. bescii* transformation, screening, purification, and sequence verification of engineered strains

To construct strain JWCB049, one microgram of M.Cbe1 methylated pDCW180 DNA was used to electrotransform JWCB017 (*C. bescii ΔpyrFA Δldh*) as described [28] and cells were plated onto solid LOD medium. Uracil prototrophic transformants were selected and used to inoculate into liquid medium for subsequent genomic DNA extraction and confirmation of the pDCW180 knock-in into the targeted chromosome via PCR screening. The insertion was targeted to base pair coordinates 962290 to 964604 in the *C. bescii* chromosome, designated CIS1 (chromosomal integration site one). Confirmed transformants were inoculated into nonselective liquid defined medium, with 40 μM uracil, and incubated overnight at 75 °C to allow loop-out of the plasmid prior to plating onto 5-FOA (8 mM) containing solid medium. After the initial screening, transformants containing the expected knock-in were further purified by one additional passage under selection on solid medium and screened a second time by PCR to check for segregation of the P_S-layer_Teth39_0206 insertion. The insertion of Teth39_0206 at the targeted chromosome region was verified by PCR amplification and sequence analysis using primers DC462, DC463, DC477, DC478 DC599, DC600, DC601 and DC602. A PCR product was generated from genomic DNA using primers DC477 and DC478 targeting outside the homologous regions used to construct the knock-in. Construction of JWCB054 was the same as JWCB049 except that pDCW183 (Table 1) was used to electrotransform JWCB017. All primers and sequences used in this study are listed in Additional file 1: Table S1.

### Preparation of cell lysates and western blotting

Five hundred mL cultures of *C. bescii* strains (JWCB017, 049, and 054) were grown to mid-log phase at 75 °C, harvested by centrifugation at 6000×*g* at 4 °C for 15 min and resuspended in Cell-Lytic B cell lysis reagent (Sigma-Aldrich, St. Louis, MO, USA). Cells were lysed by a combination of 4× freeze-thawing and sonication on ice. Protein concentrations were determined using the Bio-Rad protein assay kit (Bio-Rad Laboratories, Hercules, CA, USA) with bovine serum albumin as the standard. Cell free extracts (75 µg) were electrophoresed in 4–15 % gradient Mini-Protean TGX gels, which were either stained using Coomassie blue or were transferred to PVDF membranes (ImmobilonTM-P; EMD Millipore, Billerica, MA, USA) using a Bio-Rad Mini-Protean 3 electrophoretic apparatus. The membrane was then probed with His-tag (6xHis) monoclonal antibody (1:5000 dilution; Invitrogen, Grand Island, NY, USA) using the ECL Western Blotting substrate Kit (Thermo Scientific, Waltham, MA, USA) as specified by the manufacturer.

### Growth properties and end product analysis on various MOPS concentrations, growth curve analysis, and fermentation conditions

Growth experiments testing buffering capacity were conducted with wild type *C. bescii* in stoppered 125-mL serum bottles containing 50 mL LOD medium supplemented with 10 g/L cellobiose (catalog no. M5895; Sigma-Aldrich, St. Louis, MO, USA), 1 mM uracil and 3-morpholino-propanesulfonic acid (MOPS: catalog no. RDD003, Sigma-Aldrich, St. Louis, MO, USA) ranging from 0 to 200 mM. Duplicate bottles were inoculated with a fresh 1 % (vol/vol) inoculum and incubated at 75 °C with shaking at 150 rpm. Optical cell density was monitored using a SmartSpec™ Plus spectrophotometer (Bio-Rad Laboratories, Hercules, CA, USA), measuring absorbance at 680 nm. When testing bioconversion by engineered strains, growth was performed in LOD media supplemented with 40 mM of MOPS. Batch fermentations were performed at 75 °C in the same culture conditions, except using 10 g/L cellobiose, 20 g/L Avicel (catalog no. 11365, Fluka, Swedesboro, NJ, USA), or 20 g/L unpretreated switchgrass [sieved −20/+80-mesh fraction; washed with warm water but no additional pretreatment (note that the biomass samples were not autoclaved before use); provided by Brian Davison, Oak Ridge National Laboratory, Oak Ridge, TN, USA] as carbon sources. Fermentation products were measured for 120 h, but all fermentations were complete within 48 h.

### Analytical techniques for determining fermentation end products

On the fermentation experiments, cellobiose, glucose, acetate, lactate, and ethanol, were monitored and analyzed on an Agilent 1200 Infinity HPLC system (Agilent Tech., Santa Clara, CA, USA) fitted with a Micro-Guard Cation H+ guard column and an Aminex HPX-87H column (Bio-Rad, Hercules, CA, USA). All separations were performed under isocratic conditions with a temperature of 50 °C and a flow rate of 0.6 mL/min in 5.0 mM H_2_SO_4_, and then passed through a refractive index detector (Agilent 1200 Infinity Refractive Index Detector; Agilent Tech., Santa Clara, CA, USA). The total peak areas were integrated and compared to peak areas and retention times of known standards for each compound of interest. For H_2_ production at the end of the fermentation, H_2_ was measured using a GC8A Gas chromatograph (Shimadzu, Kyoto, Japan). Gas separations were performed on a Molecular Sieve packed column 5A (80/100 mesh; Alltech, Columbia, MD, USA) with a 70 °C oven temperature and detection with a Thermal Conductivity Detector at 120 °C. The samples were prepared from the headspace of stoppered 125-mL serum-bottle cultures using a gas-tight glass syringe (Hamilton, Reno, NV). The standards were also prepared from identical bottles with the same medium with known amounts of H_2_ in the headspace. H_2_ was calculated as moles produced per liter liquid phase to facilitate comparison to soluble fermentation products. Based on ideal gas law, pure H_2_ is assumed as 1 mol of H_2_ at 298 k (25 °C) and 1 atmosphere in 24.5 litters [54].

## Additional file

10.1186/s13068-015-0346-4 The diagram for Teth39_0206 (adhE) expression cassette integration vector in *C. bescii*.** Figure S2.** The diagram for Teth39_0218 (adhB) expression cassette integration vector in *C. bescii*.** Table S1.** List of primers used in this study.

## Abbreviations

CBP: consolidated bioprocessing
*C. bescii*: *Caldicellulosiruptor bescii*
*T. pseudethanolicus* 39E: *Thermoanaerobacter pseudethanolicus* 39E
C*. thermocellum*: *Clostridium thermocellum*
5-FOA: 5-fluoroorotic acid
ADH: alcohol dehydrogenase
ALDH: acetaldehyde dehydrogenase
AdhE: bifunctional acetaldehyde/alcohol dehydrogenase
AdhB: bifunctional acetyl CoA thioesterase/alcohol dehydrogenase
HPLC: high performance liquid chromatography
GC: gas chromatography
LDH: lactate dehydrogenase
LOD: low osmolarity defined
MOPS: 3-(*N*-morpholino) propane sulfonic acid
OD: optical density
PPP: pentose phosphate pathway
