# Supplementary material for: Cellulosic ethanol production via consolidated bioprocessing at 75 °C by engineered Caldicellulosiruptor bescii
Source: Biotechnol Biofuels. 2015 Oct 6;8:163. doi: 10.1186/s13068-015-0346-4 (PMC4595190; doi:10.1186/s13068-015-0346-4)
Supplement: Supplementary file 1 — 10.1186/s13068-015-0346-4 The diagram for Teth39_0206 (adhE) expression cassette integration vector in C. bescii. Figure S2. The diagram for Teth39_0218 (adhB) expression cassette integration vector in C. bescii. Table S1. List of primers used in this study. [file 13068_2015_346_MOESM1_ESM.docx]

**Supplementary materials**


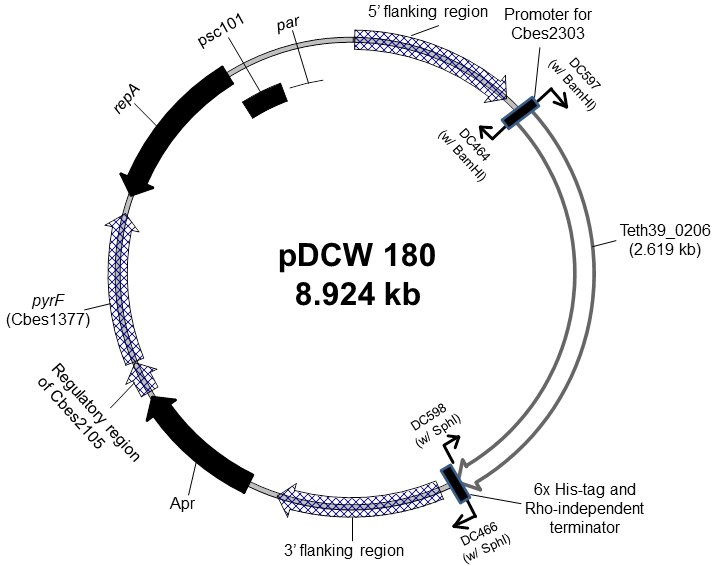


**Fig. S1. The diagram for Teth39_0206 (*adhE*) expression cassette integration vector in *C. bescii*.** Apr, apramycin resistant gene cassette; pSC101, low copy replication origin in *E. coli*; *repA*, a plasmid-encoded gene required for pSC101 replication; *par*, partition locus. Two kb flanking regions from up- and down-stream of the targeted chromosome region for homologous recombination and *pyrF* cassette for selection of uracil prototrophy are indicated. The AdhE (Teeth39_0206) expression cassette is indicated.


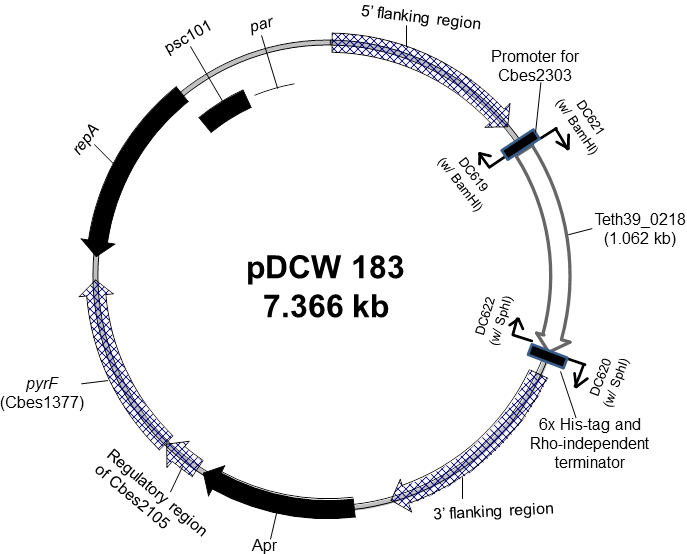


**Fig. S2. The diagram for Teth39_0218 (*adhB*) expression cassette integration vector in *C. bescii*.** Apr, apramycin resistant gene cassette; pSC101, low copy replication origin in *E. coli*; *repA*, a plasmid-encoded gene required for pSC101 replication; *par*, partition locus. Two kb flanking regions from up- and down-stream of the targeted chromosome region for homologous recombination and *pyrF* cassette for selection of uracil prototrophy are indicated. The AdhB (Teeth39_0218) expression cassette is indicated.


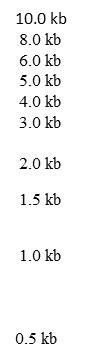

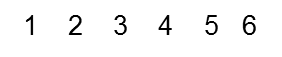

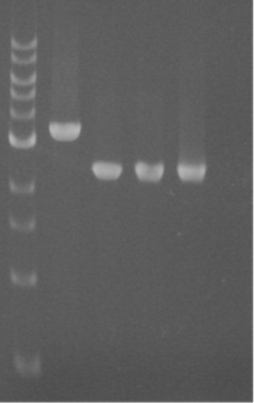


**Fig. S3. The ldh gene deletion in *C. bescii* mutant strain**s. A gel depicting PCR products amplified from the *ldh* locus; wild-type – 3.3 kb and *ldh* deletion – 2.4 kb. Lane 1, 1.0 kb DNA ladder; Lane 2, Wild-type; Lane 3, JWCB017 (*ΔpyrFA Δldh*); Lane 4, JWCB049 (*ΔpyrFA Δldh*:: P_S-layer_Teth39_0206 (*adhE*)/(ura^-^/5-FOA^R^); Lane 5, JWCB054 (*ΔpyrFA Δldh*:: P_S-layer_Teth39_0218 (*adhB*)/(ura^-^/5-FOA^R^); Lane 6, no template PCR control.

**Table S1.** List of primers used in this study.

| Primers | Sequences (5’ to 3’) | Description |
| --- | --- | --- |
| DC462 | TGCTGGCAGAGAAGAGCGAAA | To verify the targeted insertion of AdhE and AdhB cassette |
| DC463 | TCTTCATCCCAATCTTCAACTTC | To verify the targeted insertion of AdhE and AdhB cassette |
| DC464 | ACTGGATCCCTCACCAAACCTCCTTGTATGAT | To construct pDCW180 |
| DC466 | AGAGCATGCCATCACCATCACCATCACTAATAATAAAGCTGAAATAAAAGAGGGTGAGA | To construct pDCW180 |
| DC477 | TGGTTGACCAGGAGAATTTTACACA | To verify the targeted insertion of AdhE and AdhB cassette |
| DC478 | AGCAACAATCCTGCATTTGTAAG | To verify the targeted insertion of AdhE and AdhB cassette |
| DC597 | ACTGGATCCATGCCTACCTTATTACAAGAAAAAAAGGA | To construct pDCW180 |
| DC598 | AGAGCATGCTTCTCCATAGGCTTTTCTGTAAATTTCTGCA | To construct pDCW180 |
| DC599 | AATCCAGCCATTTCAGCAAT | Sequencing primer for pDCW180 |
| DC600 | GTATGCGCATCAGAACAAGC | Sequencing primer for pDCW180 |
| DC601 | TGGGTTAAAGCGTCCATACCA | Sequencing primer for pDCW180 |
| DC602 | TGTAGGTGGTGGTTCTGCAA | Sequencing primer for pDCW180 |
| DC619 | ACTGGATCC TCACCAAACCTCCTTGTATGATT | To construct pDCW183 |
| DC620 | AGAGCATGCCACCACCACCATCATCATTAATAATAAAGCTGAAATAAAAGAGGGTGAGA | To construct pDCW183 |
| DC621 | ACTGGATCCATGATGAAAGGTTTTGCAATGCTCAG | To construct pDCW183 |
| DC622 | AGTGCATGCTGCTAATATTACAACAGGTTTGATTAGG | To construct pDCW183 |
